# Addressing Determinants of Immunization Inequities Requires Objective Tools to Devise Local Solutions

**DOI:** 10.3390/vaccines11040811

**Published:** 2023-04-06

**Authors:** Siddhartha Sankar Datta, Federico Martinón-Torres, Nino Berdzuli, Niyazi Cakmak, Michael Edelstein, Simon Cottrell, Mark Muscat

**Affiliations:** 1 Vaccine-Preventable Diseases and Immunization Programme, World Health Organization Regional Office for Europe, DK-2100 Copenhagen, Denmark; 2 Translational Pediatrics and Infectious Diseases, Hospital Clínico Universitario and Universidad de Santiago de Compostela, 15706 Galicia, Spain; 3 Division of Country Health Programmes, World Health Organization Regional Office for Europe, DK-2100 Copenhagen, Denmark; 4 Azrieli Faculty of Medicine, Bar-Ilan University, Ramat Gan 5290002, Israel; 5 Vaccine Preventable Disease Programme, Public Health Wales NHS Trust, Cardiff CF10 4BZ, UK

**Keywords:** immunization, inequities, local determinants, pragmatic, operational guidance, European Immunization Agenda 2030

## Abstract

Universal immunization substantially reduces morbidity and mortality from vaccine-preventable diseases. In recent years, routine immunization coverage has varied considerably among countries across the WHO European Region, and among different populations and districts within countries. It has even declined in some countries. Sub-optimal immunization coverage contributes to accumulations of susceptible individuals and can lead to outbreaks of vaccine-preventable diseases. The European Immunization Agenda 2030 (EIA2030) seeks to build better health in the WHO European Region by ensuring equity in immunization and supporting immunization stakeholders in devising local solutions to local challenges. The factors that influence routine immunization uptake are context specific and multifactorial; addressing immunization inequities will require overcoming or removing barriers to vaccination for underserved individuals or populations. Local level immunization stakeholders must first identify the underlying causes of inequities, and based on this information, tailor resources, or service provision to the local context, as per the organization and characteristics of the health care system in their countries. To do this, in addition to using the tools already available to broadly identify immunization inequities at the national and regional levels, they will need new pragmatic guidance and tools to address the identified local challenges. It is time to develop the necessary guidance and tools and support immunization stakeholders at all levels, especially those at the subnational or local health centre levels, to make the vision of EIA2030 a reality.

## 1. Introduction

Immunization is one of the most cost-effective ways to protect populations from vaccine-preventable diseases. Consequently, ensuring universal access to immunization promotes population health and long-term prosperity [1,2]. Childhood immunization plays a key role in achieving 14 of the 17 United Nations Sustainable Development Goals (SDGs) [3], particularly SDG 3, ‘Ensure healthy lives and promote well-being for all at all ages’. However, universal access to immunization should not be interpreted as a ‘one size fits all approach’. There are population groups or individuals who will require local tailoring of services; failure to identify, acknowledge and address the barriers they face can lead to systematic inequities in immunization coverage. Immunization inequities contribute to accumulations of susceptible individuals in communities and thereby lead to outbreaks of vaccine-preventable diseases [4,5,6].

Monitoring and addressing immunization inequities are embedded in every regional and national immunization strategy in the World Health Organization (WHO) European Region (hereafter the Region). However, the countries in the Region nevertheless have difficulties in reaching and sustaining the 95% coverage target for the third dose of diphtheria-pertussis-tetanus containing vaccine (DTP3) established by European Vaccine Action Plan 2015–2020 (EVAP) [7]. The factors that influence immunization uptake and those responsible for sub-optimal vaccination coverage at subnational or national level, are multiple and often context specific [8,9,10,11]. The European Immunization Agenda 2030 (EIA2030) seeks to build better health tomorrow in the Region through stronger immunization programmes today, by ensuring equity in immunization, providing immunization across the life course and devising local solutions to local challenges [12].

It is critical that the national and subnational health systems systematically address the local factors influencing the immunization inequities, and thereby achieve and sustain high vaccination coverage in the countries. Such an approach should include a continuous process of reviewing local-level immunization coverage data, which can guide national and subnational health systems in identifying areas of sub-optimal coverage and inform national immunization policy. However, to successfully address the reasons for sub-optimal vaccination in certain areas or populations, it is critical that local-level immunization stakeholders undertake measures through use of the available tools to identify who is not vaccinated and gather information on the local drivers of and barriers to immunization uptake by the population. Tailored, local-level interventions are needed to address the identified barriers that lead to local immunization inequities. To develop these interventions, immunization stakeholders at all levels, but especially at subnational or local health centre levels, need pragmatic guidance and tools. Aligned to the core principle of EIA2030 to devise local solutions to local challenges, we outline what can be done to empower especially the subnational immunization managers and functionaries of a health system to address immunization inequities and thereby contribute to the ethos of the SDGs: “leaving no one behind”.

## 2. Discussion

### 2.1. Suboptimal Immunization Coverage and Risks of Disease

Relatively high immunization coverage in the Region over the past two decades has allowed it to sustain polio-free status since 2002 [13] and achieve significant progress in reducing the burden of measles, rubella, tetanus, diphtheria and other vaccine-preventable diseases [6]. However, routine immunization coverage still varies considerably among the Region’s 53 countries, and among different populations and districts within them. In 2021, while the regional coverage with DTP3 was 94%, 25 countries (47%) reported DTP3 vaccination coverage of more than 95%, 15 (28%) reported coverage between 90% and 95%, 11 (21%) reported coverage between 80% and 90% and 2 (4%) countries reported coverage below 80% [14]. During the same year, 13 (25%) countries reported that between 1% and 52% of their subnational administrative units attained DTP3 coverage of less than 80% [15]. A similar pattern was observed for the first dose of measles-containing vaccine (MCV1) in the Region in 2021: while the regional coverage was 94%, 28 countries (53%) reported less than 95% MCV1 coverage. These variations in immunization coverage between, and within, countries indicate that the unvaccinated and under-vaccinated populations in the Region are at high risk of vaccine-preventable diseases; where there are higher numbers of unvaccinated individuals concentrated in populations or groups, there is a higher potential for outbreaks or the re-emergence of vaccine-preventable diseases.

### 2.2. Factors Influencing Vaccination Uptake

National programmes and policymakers often attribute sub-optimal vaccination coverage to refusal to vaccinate based on concerns about vaccines, but this is only one of numerous possible contributing factors. The factors influencing vaccination coverage at local level are complex and can range from individual socio-economic conditions to issues around healthcare systems and accessibility of services [8,9,10,11]. Formative research and behavioural analysis of a Charedi Orthodox Jewish community of the London borough Hackney showed that critical issues related to sub-optimal immunization uptake were linked to access to and convenience of immunization services, both for the service providers and the population, while the assumption before the study was that under-vaccination was linked to cultural or religious anti-vaccination sentiment [16]. Several countries have shown that children living in poorer households, children born to mothers with lower levels of education and those residing in rural areas are more likely to be left behind in immunization uptake [17]. Vaccination coverage has also been found to be lower in specific population groups like asylum seekers, refugees, migrants and deprived communities in comparison to the general population [17,18,19]. In addition, studies have also demonstrated the role ethnicity can play as a cultural factor in influencing completion of scheduled vaccination doses [20,21]. Indeed, certain ethnic communities such as Roma and Sinti have often been disproportionately affected by outbreaks of measles [22]. Careful consideration of factors such as area of residence, living conditions and characteristics including age, gender, economic status, ethnicity, religion, migration status, education or disability will help national and subnational immunization programme managers to develop appropriate immunization delivery strategies that yield more equitable uptake.

### 2.3. Immunization Inequity and Regional Immunization Strategies

EVAP suggested that countries in the Region ensure that every individual is eligible to receive all appropriate vaccines, irrespective of their geographic location, age, gender, educational level, socioeconomic status, ethnicity, nationality or religious or philosophical affiliation [7]. EIA2030 builds on the successes and lessons learned through implementation of EVAP, with a vision and strategy for achieving the full benefits of immunization in the Region for the next decade, seeking to attain stronger and more resilient immunization programmes by focusing on three key principles: ensuring equity in immunization, providing immunization across the life course and devising local solutions to local challenges [12]. By ensuring that national immunization strategies have an equity-based approach to reach unvaccinated and under-vaccinated populations, EIA2030 aims to address the inequities in immunization coverage between and within countries through the use of innovative programming, better understanding of the concept of immunization equity and local-level interventions to identify and address barriers.

### 2.4. Identifying and Addressing Immunization Equity

In addition to determining the administrative areas of sub-optimal coverage by evaluating the routinely reported annual immunization coverage data, it is critically important for subnational immunization stakeholders to identify which populations groups in these areas have lower vaccination uptake and understand why they are not vaccinated. These will allow the stakeholders at the local level to devise tailored strategies to improve the vaccination coverage and thereby also reduce inequity.

Inequities are reflective of population groups in communities being left behind. As a starting point, understanding immunization inequities will require good-quality, robust disaggregated immunization coverage data at every level of a health system together with systematic monitoring. Since 2016, tools [23,24,25] developed by WHO have supported countries to illustrate socio-economic, demographic and geographic variation in uptake, enable comparisons of data about immunization (and other health parameters) within and across countries and devise analytical approaches to determine the factors associated with immunization coverage. Every country, through its network of national and sub-national immunization programmes, should examine its local-level immunization uptake data to identify the presence or absence of inequities. Once population groups with low immunization coverage are identified, understanding the contextual issues they face is the next step toward improving vaccine equity and averting future outbreaks, especially among those who are also often at higher risk of severe but preventable outcomes. During EVAP implementation, the WHO Regional Office for Europe supported countries in the Region through implementation of the “Tailoring immunization Programmes” (TIP) initiative to diagnose vaccination barriers and motivators in populations with low vaccination coverage and design tailored interventions to address these barriers [26]. Whilst TIP provided a framework for countries to understand the perspective of populations with low vaccination coverage, an external review of TIP implementation in four countries in the Region in 2016 suggested that the efforts should go beyond identification of susceptible groups and diagnosis of challenges [27].

The organization and delivery of vaccination programmes vary widely in the Region, [28] thus preventing or reducing inequities in immunization coverage will inevitably require local-level tailoring of resources or service provision for underserved individuals or populations to overcome or remove barriers to vaccination. Immunization programme monitoring must now go beyond national or regional estimates of immunization coverage and additional efforts should be made to understand how vaccination uptake varies according to socioeconomic, demographic and geographic factors within a country. It is time that simple and pragmatic guidance and tools are developed for the immunization stakeholders at all levels especially for those at the local health facility levels, to identify and understand barriers to immunization uptake. This will pave the way for the subnational immunization managers to develop and implement successful interventions addressing these immunization inequities coupled with periodic evaluation of the targeted interventions and mid-course corrections, if need be.

### 2.5. Characteristics of Guidance and Operational Frameworks

Across the Region, while countries and immunization programmes vary widely in their immunization service delivery systems, they are also at different stages of recognizing, considering and addressing issues of immunization inequity. Thus, guidance and tools should contain pragmatic operational frameworks for all levels of the healthcare system, from the local health facility to the Ministry of Health.

The operational frameworks should be cohesive, implementable and relevant to the immunization stakeholders at national, sub-national, regional and local health facility levels. With a primary focus on “how to”, pragmatic guidance and tools should focus on the following stakeholders to achieve a local demonstrable impact on immunization inequity:

- decision- and policy-makers who can advocate for equity within immunization programmes, to ensure the required political support is maintained to tackle immunization inequity as part of the broader heath policy agenda;
- immunization programme managers at the national and subnational levels who are developing and implementing immunization strategies, to embed equity in planning, delivery and monitoring of the immunization programmes;
- immunization programme managers and their staff who are tasked with identifying, addressing and monitoring health inequities, to gather and use information on local determinants of inequities and make informed decisions on interventions.

The TIP tools and guidance developed by the WHO Regional Office for Europe are already available to support the immunization programme managers and their staff in identifying and characterizing population groups with lower immunization uptake and to diagnose vaccination behaviour barriers, system barriers and motivators.

An operational guide which contains pragmatic, cohesive and implementable operational frameworks relevant to the immunization stakeholders for all levels of health system and relevant, simple and action-oriented tools to address immunization inequity at the local health facility level is being developed by the WHO Regional Office for Europe in consultation with the countries in the Region, to empower subnational immunization managers to address determinants of immunization inequities closer to the location where the immunization inequity exists. Together with developing these pragmatic tools to address immunization inequity at the local health facility level, the WHO Regional Office for Europe will support capacity building of the national and subnational immunization programme managers on the use of these tools. Successful implementation of interventions to reduce or prevent immunization inequities at the local levels will require multipronged actions involving the stakeholders who have roles in vaccine programme planning and delivery and those involved in advocating for immunization equity in underserved groups. Only through developing and implementing robust local-level interventions will countries in the Region be able to achieve EIA2030′s strategic priority on immunization equity: namely, ensuring that routine immunization coverage is high in every community and that all individuals have equitable access to and adequately utilize all vaccines in national immunization schedules.

Addressing immunization inequity between population groups within a country will have an impact on the healthcare delivery and population health as a whole. Like immunization coverage, achieving equity should be viewed as a systematic and continuous process.

## 3. Conclusions and Future Directions

Attaining and maintaining high and equitable immunization coverage in every subnational administrative unit in the Region will contribute to better population health by expanding protection to those who are currently at most risk of acquiring vaccine-preventable diseases. Such an approach will reduce the risk of outbreaks of vaccine-preventable diseases and help address wider inequities in health. Achieving the health-related SDGs requires urgent attention to close the health inequity gap through collection and use of data and information at the local health facility level. Reducing inequity should be embedded as a core aim of national immunization programmes; accordingly, reducing local level inequalities in immunization service delivery and utilization must be a critical cornerstone of every national immunization strategy. Identifying, addressing and monitoring inequity within immunization programmes should become a systematic and ongoing process, with solutions tailored to the context of each country and population. A simple, effective, action-oriented and pragmatic operational guide designed especially for subnational immunization programme managers with appropriate linkages to all levels of health systems is critical in achieving the strategic immunization priorities outlined in EIA2030. This will ensure everyone everywhere in the Region reaps the benefits of the vaccines in national immunization schedules.

## Data Availability

Data sharing not applicable.
